# Formulation of cost-effective medium and optimization studies for enhanced production of rapamycin

**DOI:** 10.1186/s12934-023-02201-3

**Published:** 2023-09-20

**Authors:** Sanjeev. K. Ganesh, Subathra Devi C

**Affiliations:** School of Bio Sciences and Technology, Vellore Institute of Technology, Vellore, 632014 Tamil Nadu India

**Keywords:** Rapamycin, *Streptomyces hygroscopicus*, Cost-effective medium, Ultraperformance liquid chromatography (UPLC), Response surface methodology (RSM)

## Abstract

**Background:**

Enhancing rapamycin production using a cost-effective medium is crucial for wider accessibility, reduced manufacturing costs, sustainable pharmaceutical practices, and advancements in therapeutic applications. It promotes global health, biotechnological innovation, research collaboration, and societal well-being through affordable and effective treatments. This study focuses on the development of a novel cost-effective production medium for the synthesis of rapamycin from *Streptomyces hygroscopicus*.

**Results:**

In the initial screening, more rapamycin production was observed in medium A. Initially, the organism produced 10 µg/mL rapamycin. Based on the OFT results, a novel cost-effective medium composition was designed, incorporating soyabean, sugarcane juice, and dried tomato components. Using RSM, soyabean and tomato was found to be more significant in rapamycin production than sugarcane. In the optimized medium, the production of rapamycin increased significantly to 24 µg/mL. Furthermore, a comparative analysis of the growth kinetics between the production normal medium (referred to as production medium A) and the newly optimized cost-effective production medium revealed that the optimized cost-effective production medium significantly enhanced the production of rapamycin.

**Conclusion:**

Overall, this study demonstrates the successful development of a cost-effective production medium for rapamycin synthesis from *S. hygroscopicus*. The findings highlight the potential of using a cost-effective medium to enhance the production of a valuable secondary metabolite, rapamycin, while reducing production costs.

**Supplementary Information:**

The online version contains supplementary material available at 10.1186/s12934-023-02201-3.

## Background

Rapamycin is exceedingly essential worldwide due to its diverse applications. It functions as an immunosuppressive drug for organ transplantation, has potential in cancer treatment, and has implications in antiaging research. Its versatility makes it a valuable compound in various aspects of medicine, science, and sustainability [1]. Rapamycin is produced by the bacterium *S. hygroscopicus* and belongs to a class of compounds called macrolide antibiotics. The mechanism of rapamycin production in *S. hygroscopicus* involves a complex biosynthetic pathway. It begins with the activation of the rapamycin biosynthetic gene cluster and the incorporation of amino acids L-pipecolic acid and L-lysine. Polyketide synthesis resulting in the formation of a polyketide chain. Tailoring reactions modify the polyketide chain, and cyclization and rearrangement reactions form the macrocyclic structure of rapamycin. [2, 3].

A key regulator of cell growth and metabolism in human cell is mammalian target of rapamycin complex 1 (mTORC1). When rapamycin binds to 12-kDa FK506-binding protein (FKBP12), a complex is created that blocks mTORC1 and suppresses protein synthesis, cell growth and proliferation, and autophagy [4–7]. Inhibiting mTORC1 has an immunosuppressive effect by reducing T-cell activation and proliferation [8, 9]. Rapamycin’s ability to modify mammalian target of rapamycin (mTOR) signaling has projected benefits in transplantation, cancer therapy, and age-related disorders [10–12], even though the exact mechanisms and downstream consequences are still not completely explored. Inhibition of mTOR signaling leads to direct antitumor effects, such as the inhibition of cancer cell proliferation and induction of apoptosis. Additionally, rapamycin interferes with angiogenesis, the process by which tumors form new blood vessels, thereby limiting the nutrient supply to the tumor and impeding its growth [13–15].

The production of rapamycin faces several difficulties that hinder its efficient and cost-effective manufacturing. A challenge in rapamycin production is that the synthesis of rapamycin involves multiple enzymatic reactions and requires the coordinated action of various enzymes. This complexity makes it difficult to engineer and optimize the production process [16]. Additionally, rapamycin production often yields low quantities, posing challenges for large-scale production. Factors such as suboptimal growth conditions, inefficient enzyme expression, and metabolic limitations within the producing microorganism contribute to these low yields [17]. Another difficulty lies in the genetic manipulation of the producing microorganism, as it requires a deep understanding of the biosynthetic pathway and genetic engineering techniques to achieve stable overexpression of key biosynthetic genes without compromising the microorganism’s growth and viability [18]. Furthermore, optimizing fermentation conditions, including pH, temperature, and nutrient availability, is crucial but challenging due to the delicate balance required for optimal microbial growth and rapamycin biosynthesis. Downstream processing, involving the extraction and purification of rapamycin from the fermentation broth, is also complex and time-consuming. It requires disrupting cells, separating the target compound from other cellular components, and multiple purification steps. Finally, the cost of rapamycin production is a significant barrier, driven by the complexity of the process, low yields, and the need for specialized equipment and skilled personnel. Overcoming these difficulties requires continuous research and development efforts to optimize fermentation conditions, improve genetic manipulation techniques, enhance downstream processing methods, and explore more cost-effective production strategies. The current study was aimed to develop a novel cost-effective medium for the production of rapamycin by employing Box-Behnken statistical analysis (BBS). The primary objective was to identify a suitable production media for rapamycin using laboratory chemicals and reagents, followed by optimization of the media components using one factor at a time (OFT) analysis method. The second objective was to identify cost effective, natural alternatives for the media supplementation, followed by standardization of the media using RSM. A comparison of the growth kinetics and rapamycin production in both the media was performed to evaluate the economic feasibility of the newly formulated production media. By identifying and utilizing the most economical sources of nutrients, the study expects to significantly reduce the production costs associated with rapamycin medium formulation.

The novelty of this research lies in its economic feasibility when compared to the current scenario, where it costs approximately 4.4 USD for 1 L production media. This high cost is due to the use of commercially purchased fructose, mannose, soya peptone, ammonium sulphate, lysine, casein and trace elements. Whereas, the newly formulated production media substituted with natural ingredients, costs around 0.32 USD. This could bring about a drastic decline in the overall production cost of rapamycin.

## Results

### Comparison of rapamycin production in different media

In comparison with medium B and medium C, medium A showed greater activity against *Candida albicans.* Production medium A showed a zone of inhibition of 22 mm, followed by production medium B (18 ± 2.3 mm) and production medium C (9 ± 1.6 mm). Production medium C showed the lowest production of rapamycin. Production medium A was found to be optimum for rapamycin production using *S. hygroscopicus* and was selected for further study. Figure S1 represents the anti candida activity of rapamycin extracted from production medium A, medium B and medium C.

### Quantification of rapamycin content

**Fig. 1:**
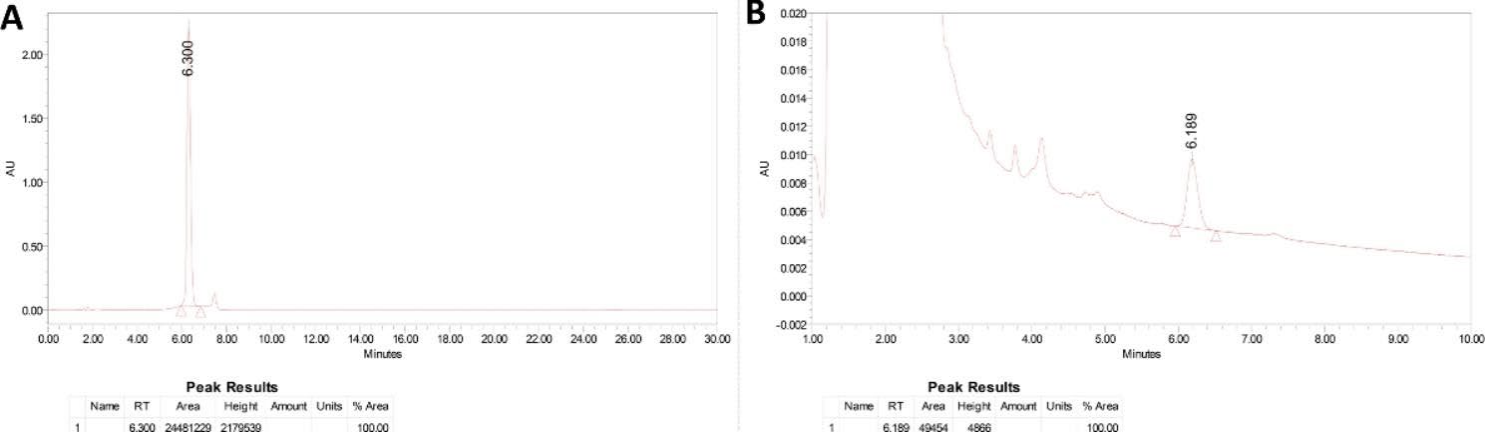
Standard rapamycin chromatogram (**A**) and chromatogram of rapamycin extracted from *Streptomycetes hygroscopicus* (**B**)

The presence of rapamycin was analyzed using UPLC. The toluene extract of medium A showed a peak at a retention time of 6.189 min. A similar peak was obtained at 6.300 min in the rapamycin standard (Fig. 1). Thus, the presence of rapamycin in medium A was confirmed. The concentration of rapamycin was determined from the standard curve (R^2^ = 0.999). From the linear regression equation, the concentration of rapamycin was estimated to be 10.8 µg/mL.

### Determining the time-dependent correlation of rapamycin production with anti-candida activity

**Fig. 2:**
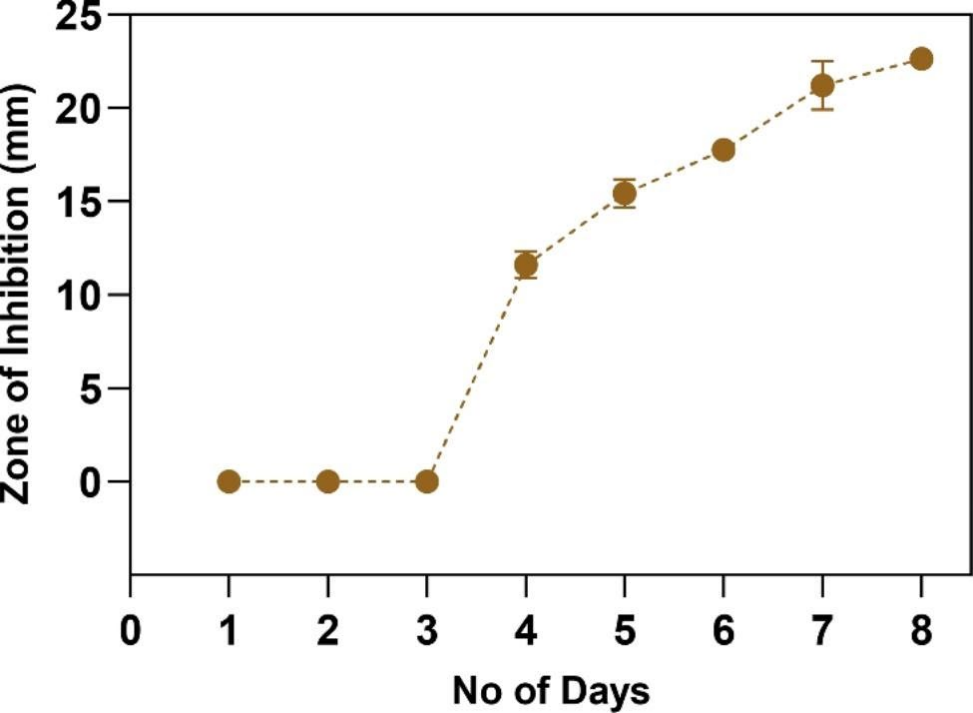
Time-dependent changes in the zone of inhibition against *C. albicans* based on rapamycin production for a period of 8 days

A detectable increase in cell density was obtained from the 3^rd^ day of inoculation. The increase in rapamycin production was determined by comparing the zones of inhibition on each day. On the 4^th^ day of incubation, a zone of inhibition measuring 11.6 ± 1.3 mm was observed against *C. albicans.* Thus, rapamycin production started on the 4^th^ day. An increase in rapamycin production was observed gradually with respect to time.The maximum zone of inhibition was observed on the 7^th^ (22 ± 2.5 mm)and 8^th^ (23 ± 0.6 mm) days of incubation. Here, there was no significant increase in production on day 8 compared to that on day 7. Hence, it was found that the maximum production was attained during the 7^th^ and 8^th^ days of incubation. Figure 2 represents the zone of inhibition with respect to the production of rapamycin by *S. hygroscopicus*.

### Media optimization

#### Carbon source

The impact of different carbon sources on rapamycin production was investigated by assessing the zone of inhibition against *C. albicans*. Each carbon source was individually tested, and the resulting zones of inhibition were measured to evaluate their effects on rapamycin synthesis.

Among the tested carbon sources, sorbitol demonstrated a moderate effect on rapamycin production, as indicated by a zone of inhibition measuring 11.3 ± 2.3 mm. Fructose, on the other hand, exhibited a significantly larger zone of inhibition, measuring 20.6 ± 1.4 mm, suggesting a positive influence on rapamycin synthesis. Dextrose, although still inhibitory, demonstrated a smaller zone of inhibition of 17.6 ± 1.6 mm compared to fructose. Sucrose, another carbon source tested, resulted in a zone of inhibition measuring 14.3 ± 1.3 mm, indicating a moderate effect on rapamycin production. Xylose, similar to fructose, demonstrated a larger zone of inhibition of 24.6 ± 1.6 mm, suggesting its potential to enhance rapamycin production. Lactose, the final carbon source tested, exhibited the smallest zone of inhibition measuring 10 ± 1 mm, indicating a minimal effect on rapamycin synthesis. These results highlight the varying impacts of different carbon sources on rapamycin production. Fructose and xylose were found to be the optimum carbon sources, as they exhibited maximal zones of inhibition against *C. albicans* compared to other tested sources.

### Nitrogen source

Among the tested nitrogen sources, soya peptone medium exhibited the maximum zone of inhibition, measuring 26.3 ± 7 mm. This indicated a significant positive effect on rapamycin production when using soya peptone as a nitrogen source. In the media supplemented with ammonium phosphate, a zone of 16.3 ± 1.7 mm was observed, which indicated the moderate effect of the compound on rapamycin biosynthesis. Additionally, in the media supplemented with ammonium sulfate and beef extract, smaller zones were observed (15.6 ± 2.4 mm and 15.3 ± 2.7 mm, respectively), substantiating their minimal impact on rapamycin synthesis. In contrast, no zone was observed for the medium containing peptone, yeast extract, ammonium nitrate, casein, urea, ammonium chloride, and potassium nitrate. These nitrogen sources showed no significant effect on rapamycin production.

Based on the observations, soya medium was found to be an optimum nitrogen source for rapamycin production. Other tested nitrogen sources (ammonium phosphate, ammonium sulfate, beef extract, peptone, yeast extract, ammonium nitrate, casein, urea, ammonium chloride, and potassium nitrate) did not exhibit substantial inhibitory effects on the growth of *Candida* and hence could not be regarded as a good choice of nitrogen source for rapamycin production.

### Amino acids

Lysine supplementation exhibited the largest impact, resulting in a zone of inhibition measuring 42.3 ± 1.7 mm. This indicated that lysine significantly enhanced rapamycin production. Additionally, tyrosine supplementation produced a zone of inhibition measuring 37.3 ± 7 mm, highlighting its positive influence on rapamycin synthesis. Additionally, tryptophan and glutamine supplementation produced zones of 36.3 ± 1.7 mm and 36 ± 2 mm, respectively, suggesting their beneficial effects on rapamycin production. Supplementation with glycine led to a slightly smaller zone of inhibition of 34 ± 1 mm, indicating a favorable impact on rapamycin synthesis. Valine and aspartate supplementation resulted in zones of inhibition of 33 ± 2 mm and 31.3 ± 1.7 mm, respectively, suggesting a moderate positive effect. On the other hand, ornithine supplementation showed a relatively smaller impact, with a zone of inhibition measuring 26.6 ± 1.4 mm. Serine supplementation exhibited a further smaller effect, with a zone of inhibition of 25 ± 1.6 mm. In contrast, neither butyric acid nor cysteine supplementation induced any zone of inhibition, suggesting their inhibitory role in rapamycin production.

Hence, lysine, tyrosine, tryptophan, and glutamine demonstrated significant positive effects on rapamycin production based on the zones of inhibition. Ornithine, valine, and aspartate also contributed to the enhancement of rapamycin synthesis. However, glycine and serine exhibited relatively smaller impacts, while butyric acid and cysteine completely inhibited rapamycin production.

#### NaCl concentration

Supplementation with 1% NaCl in the medium resulted in a zone of inhibition measuring 40.6 ± 1.4 mm. This finding suggested that 1% NaCl positively affects rapamycin production. In contrast, medium supplemented with 1.5% NaCl exhibited a smaller zone of inhibition, measuring 32.5 ± 2.7 mm. Although this concentration indicated some inhibitory effect, it was less significant compared to that of 1% NaCl supplementation. No zones of inhibition were observed in the medium supplemented with 0% and 0.5% NaCl. These results suggested that lower NaCl concentrations may have little or no impact on rapamycin production, as indicated by the absence of inhibitory effects against *C. albicans*. Furthermore, supplementation of the medium with 2% NaCl resulted in a zone of inhibition measuring 26.3 ± 1.7 mm. This suggested that higher NaCl concentrations may have a further diminishing effect on rapamycin production.

These findings suggest that a NaCl concentration of 1% promotes enhanced rapamycin production, while higher concentrations (1.5% and 2% NaCl) give reduced production.

### pH

**Fig. 3:**
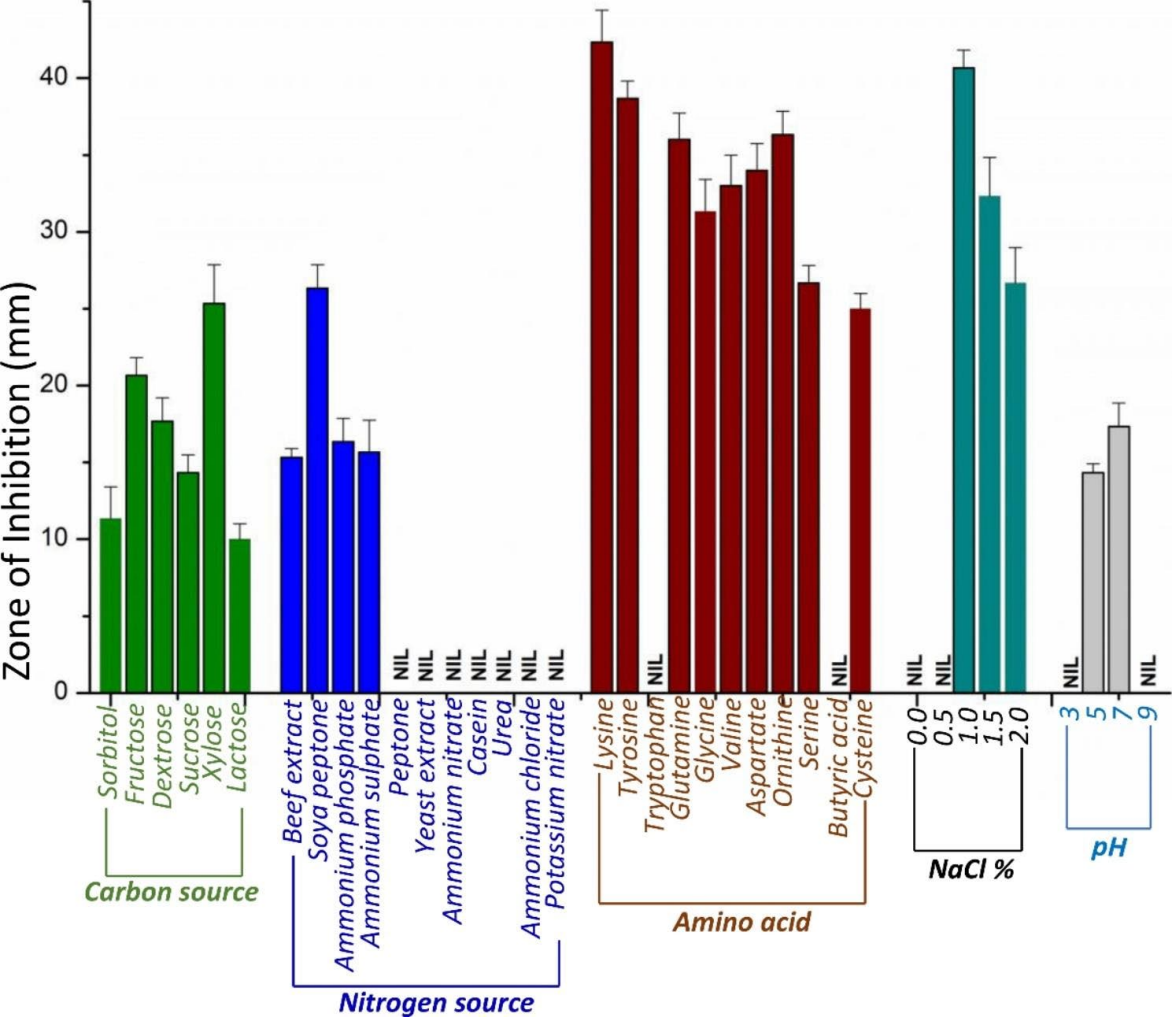
Zone of inhibition against *C. albicans* in medium supplemented with different carbon and nitrogen sources, amino acids, NaCl concentration and pH

In the pH 7 medium, a large zone of inhibition measuring 17.3 ± 1.2 mm was observed and indicated the enhanced rapamycin production. On the other hand, in pH 5 medium, a smaller zone of inhibition measuring 14.3 ± 1.8 mm was observed, suggesting a slight inhibitory effect on rapamycin production in acidic pH compared to pH 7. Interestingly, no zone of inhibition was found in the medium with pH 3 and 9. This indicated that highly acidic and alkaline pH values are not favorable for rapamycin production. Anti-candida activity based on different carbon sources, nitrogen sources, amino acids, NaCl concentration and pH is illustrated in Fig. 3.

### Screening for cost-effective medium

The zone of inhibition against *C. albicans* was evaluated to formulate a cost-effective production medium for rapamycin production. Medium supplemented with sugar cane juice (substitute for xylose) showed the highest zone of inhibition of 38 ± 2 mm. Corncob and rice straw exhibited relatively smaller zones (22 ± 0.9 and 18 mm, respectively), while no inhibitory zones were observed in bagasse supplemented medium. Among the nitrogen sources, medium substituted with soya bean demonstrated a zone of inhibition of 42 ± 1.2 mm, whereas media with *Pisum sativum* leaves and grass exhibited no zones. A 45 ± 1.5 mm zone was obtained in the media in which lysine was substituted with tomato. Hence, incorporation of sugar cane juice and soybean as carbon and nitrogen sources could significantly enhance rapamycin production in comparison to the other substituent components used. Additionally, tomato is a promising lysine source for the production medium.

### Optimization of cost-effective medium

Box–Behnken statistical analysis was used to determine the optimum effective formulation for the enhanced production of rapamycin. A total of 17 runs were performed with different concentrations of soya, tomato, and sugarcane (Table 1). The maximum rapamycin production was observed at 24.6 µg/mL in run 6. The statistical method suggested that the linear model was a significant fit with the highest *F* value (99.59). The system generates a second-order polynomial equation for rapamycin production:

Rapamycin production (µg mL^− 1^) = 19.37 ± 0.5543 A ± 3.40B ± 1.66 C.

where A, B, and C represent the coded terms for the three test variables sugarcane, tomato, and soya, respectively.

**Table 1:** Concentration of rapamycin produced in each run

Run	Factor 1 A: sugarcane (mL L^− 1^)	Factor 2 B: Tomato (g L^− 1^)	Factor 3 C: Soya (g L^− 1^)	Response 1 Rapamycin (µg mL^− 1^)
1	200	12.5	20	21.27
2	100	20	12.5	21.812
3	150	12.5	12.5	19.874
4	200	5	12.5	16.424
5	150	5	5	12.95
6	150	20	20	24.6
7	100	5	12.5	15.42
8	150	12.5	12.5	19.87
9	150	20	5	20.55
10	100	12.5	20	19.963
11	100	12.5	5	18.069
12	150	12.5	12.5	19.62
13	150	5	20	17.965
14	200	12.5	5	18.984
15	200	20	12.5	23.02
16	150	12.5	12.5	19.76
17	150	12.5	12.5	19.07

**Fig. 4:**
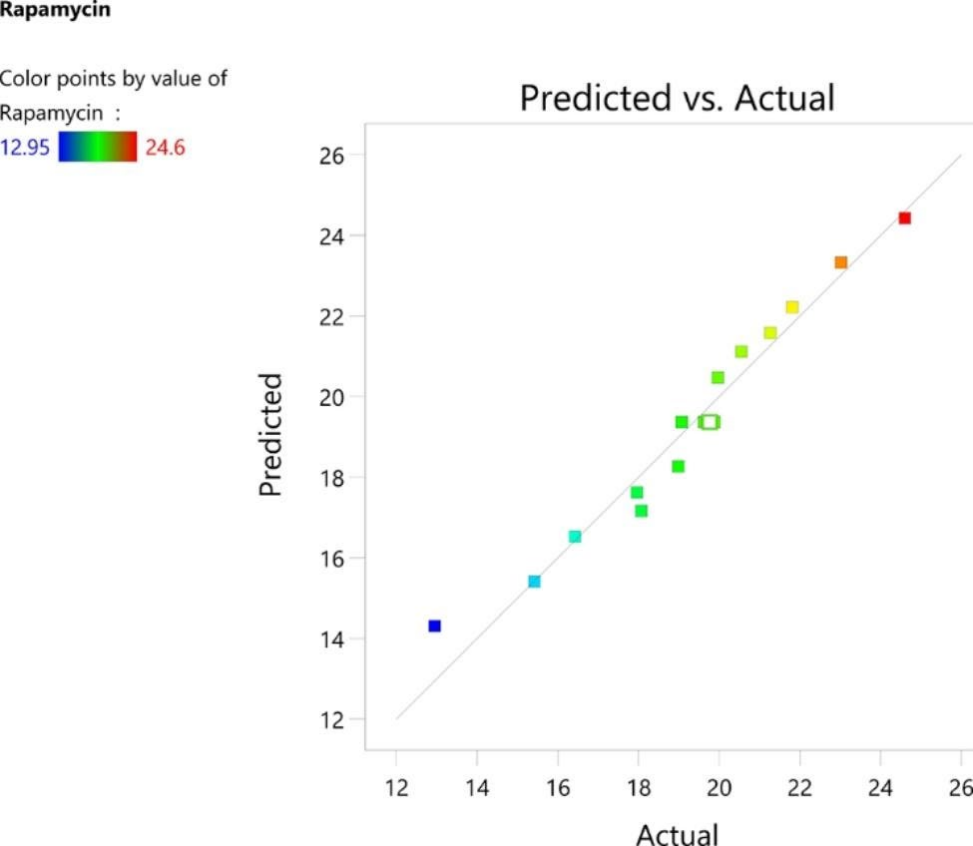
The actual values aligned with the predicted values, signifying the accuracy of the experiment

**Fig. 5:**
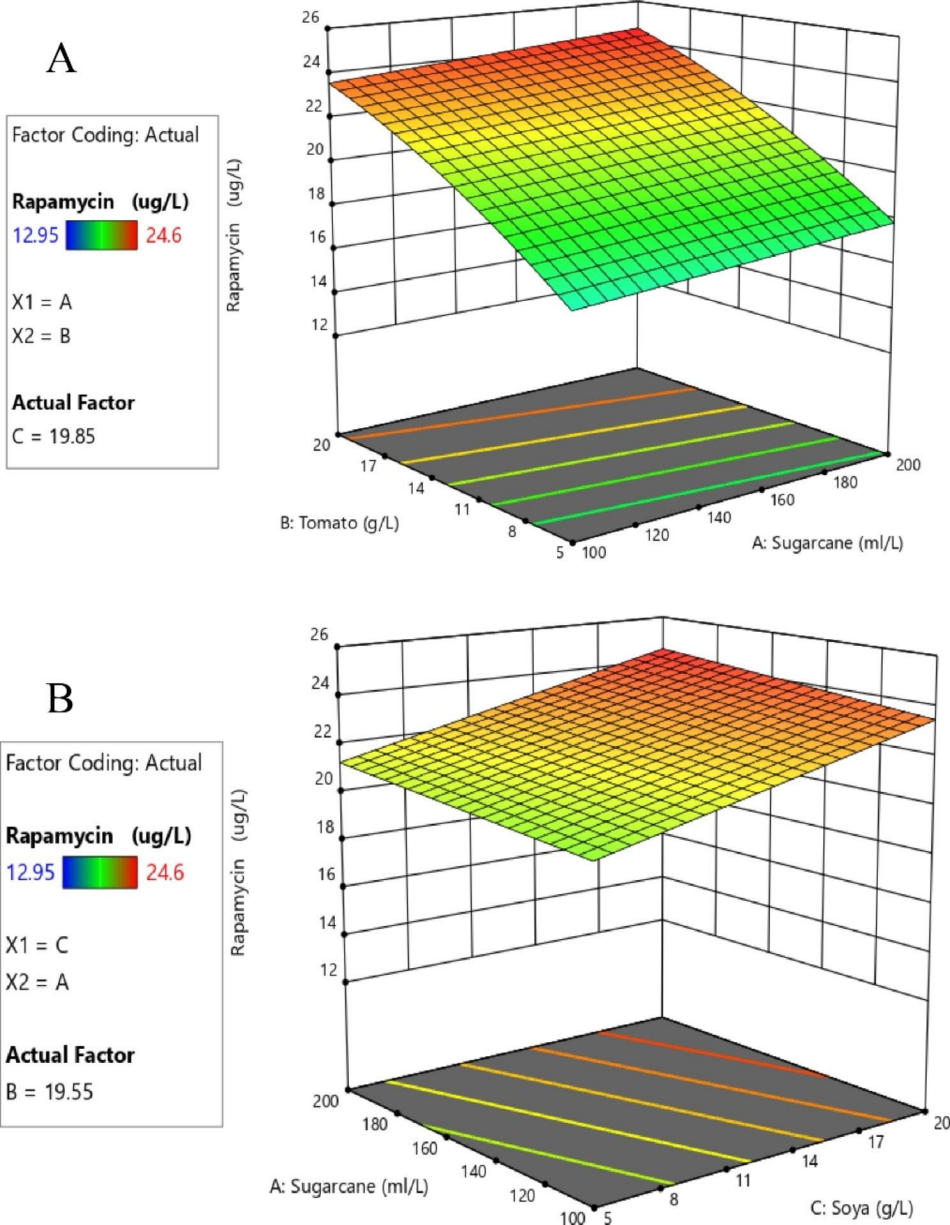
**A**: 3-D interactions between tomato and sugarcane for medium optimization. **B**: 3-D interactions between sugarcane and soya for medium optimization

ANOVA demonstrated that *the F* test obtained the highest significance (p < 0.0001) for the regression. The R^2^ value of the fit statistic is 0.9583. The difference between the adjusted R^2^ (0.9487) and predicted R^2^ (0.9192) values was less than 0.2, which indicated that the value was fit for analysis. This implied that 99.59% of the response variance could be explained well and that a 0.41% chance of variability occurred during the experiments. Based on the *p* values, multiple regression analysis demonstrated that the terms (A, B and C) in the investigated model were significant (Table 2). Based on *the F* value, the analyzed model indicated that B (*F* value = 236.61) had a greater impact on the enhanced production of rapamycin than A and C. Variable A (*F* value = 6.27) had the least impact on the production of rapamycin compared to variable C (F value = 55.99). Figure 4 illustrates the predicted vs. actual graph, experimental values, and the predicted values aligned with each other, indicating the accuracy of the experiment. Figure 5 presents a three-dimensional (3D) graph representing the optimum concentration of each variable for rapamycin production. The 3D graph represents the significant impact of different variables on the production of rapamycin, either separately or in combination. The correlation between different concentrations of sugarcane (198.8 mL L^− 1^) and tomato (19.85 g L^− 1^) at a constant soya concentration (19.55 g L^− 1^) is represented in Fig. 5A. At this particular concentration, maximum rapamycin production of 24.68 µg/mL was attained. Figure 5B elaborates the correlation between the different concentrations of sugarcane (199.05 ml L^− 1^) and soya (19.85 g L^− 1^) at a constant tomato concentration (19.85 g L^− 1^) and maximum rapamycin production of 24.65 µg/mL was obtained. Based on the actual vs. prediction graph and 3D graph (Figs. 4 and 5), the optimum concentrations of sugarcane, tomato and soya needed for maximum production were found to be 150 g L^− 1^, 19.85 g L^− 1^ and 19.85 g L^− 1,^ respectively.

**Table 2:** ANOVA table for the linear model

Source	Sum of Squares	df	Mean Square	F value	p value	
**Model**	117.02	3	39.01	99.59	< 0.0001	significant
A-Sugarcane	2.46	1	2.46	6.27	0.0147	
B-Tomato	92.64	1	92.64	236.51	< 0.0001	
C-Soya	21.93	1	21.93	55.99	< 0.0001	
**Residual**	5.09	13	0.3917			
Lack of Fit	4.46	9	0.5161	4.61	0.0775	Not significant
Pure Error	0.4473	4	0.01118			
**Cor Total**	122.11	16				

**Comparative analysis of the production of rapamycin in production medium A and RSM optimized medium over time**.

**Fig. 6:**
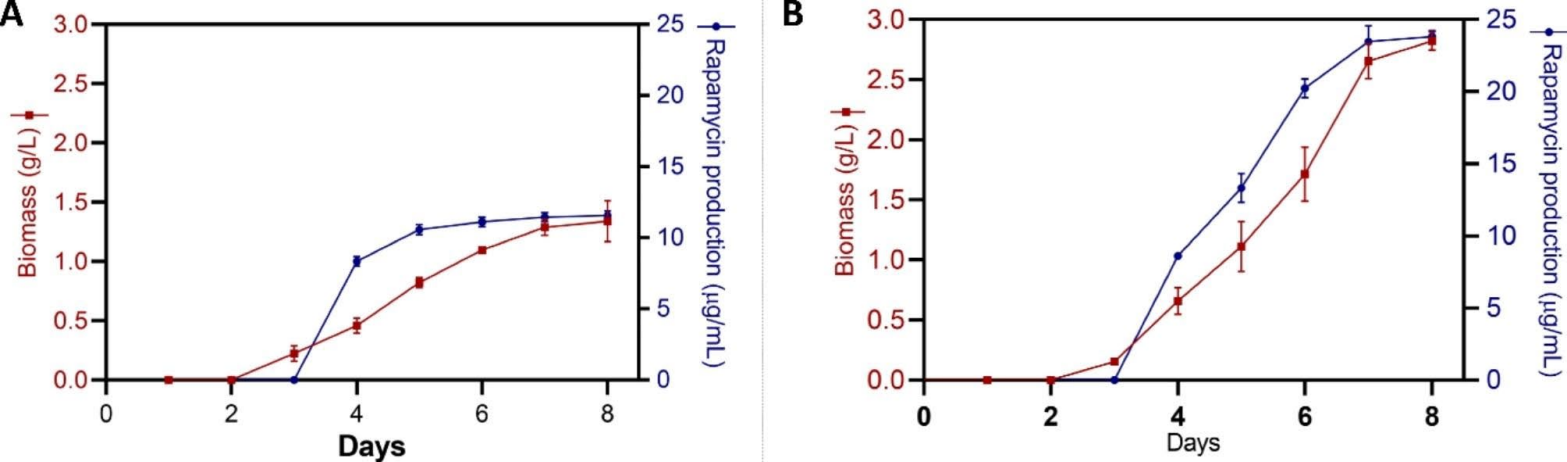
Rate of rapamycin production and increase in biomass in (**A**) medium A and (**B**) cost-effective medium over time

The growth kinetics and rapamycin production by *S. hygroscopicus* were investigated in two different production media. Figure 6 illustrates the comparative analysis of rapamycin production and biomass growth in production medium A and cost effective medium. In production medium A, no significant cell growth was observed during the first two days. On the third day, 0.244 ± 0.055 g/L cell growth was observed, but no rapamycin production was detected. However, on the fourth day, rapamycin production of 8.52 µg/mL was observed. Subsequently, there was a gradual increase in rapamycin production on the fifth, sixth, seventh and eighth days (10.84 µg/mL, 11.17 µg/mL, 11.44 µg/mL and 11.47 µg/mL, respectively). The total biomass content increased exponentially from the 4^th^ day to the 7^th^ day (0.459 ± 0.69 g/L to 1.288 ± 0.149 g/L). The biomass content recorded on the 7^th^ and 8^th^ days (1.288 ± 0.057 g/L and 1.34 ± 0.2 g/L) did not show significant variation.

A similar growth pattern was initially exhibited in the RSM-optimized cost-effective production medium, with no cell growth observed in the first two days. On the third day, there was a small amount of cell growth but no rapamycin production. However, starting from the fourth day, rapamycin production was detected at a significant concentration of 8.59 µg/mL. Notably, in this optimized medium, rapamycin production showed a substantial increase over time. On the fifth day, the concentration reached 13.5 µg/mL, and it further increased to 20.4 µg/mL, 23.76 µg/mL, and 24.1 µg/mL on the sixth, seventh, and eighth days, respectively. The biomass content was found to increase from the 3^rd^ day (0.270 ± 0.17 g/L) onward, and an exponential increment up to the 7^th^ day (2.65 ± 0.16 g/L) was noted. No significant differences in biomass content were observed between the 7^th^ (2.65 ± 0.16 g/L) and 8^th^ days (2.821 ± 0.79 g/L).

These results indicate that the RSM-optimized cost-effective production medium significantly improved the production of rapamycin compared to medium A. The optimized medium allowed higher final concentrations. This suggests that the formulation of the cost-effective medium may have provided necessary nutrients or favorable conditions for the *S. hygroscopicus* strain to enhance rapamycin production.

## Discussion

In the present study, medium A exhibited higher rapamycin production due to its diverse carbon and nitrogen sources. Additionally, in medium A, trace elements acted as cofactors, enhancing metabolic activity and increasing rapamycin production [19]. Media B and C had limited substrates, resulting in lower yields. In the previous studies, researchers have commonly used reverse phase high-performance liquid chromatography (RP-HPLC) for rapamycin estimation and quantification [20]. In the current study, ultra-performance liquid chromatography (UPLC) was used to analyze the rapamycin concentration in the medium. The adoption of UPLC in our research demonstrates the continual advancements in analytical techniques for rapamycin analysis. The use of UPLC offers a more efficient and accurate method for rapamycin estimation.

The current research investigated the impact of different carbon sources, nitrogen sources, amino acids, NaCl concentrations, and pH on rapamycin production. The results revealed that fructose and xylose had a positive influence on rapamycin production, while soya peptone was the most effective nitrogen source. Among the amino acids, lysine significantly enhanced rapamycin synthesis. The current observations align with the findings of Abdel and Dutta et al., who reported that fructose, soya peptone and lysin-HCl have a positive effect on enhancing the production of rapamycin [20, 21]. In the present study, xylose-supplemented medium exhibited a slight increase in rapamycin production compared with fructose supplemented medium. Previous reports align with the current finding that supplementing the medium with lysine enhances rapamycin production [22]. Lysine is an important precursor for the synthesis of L-pipecolic acid, which plays a crucial role in the structure of rapamycin. Increasing the availability of lysine in the medium significantly increases the availability of free L-pipecolic acid in the microbial cell factory of *S. hygroscopicus* [23]. This, in turn, leads to enhancement in rapamycin production.

Furthermore, to identify cost-effective alternatives for media components, natural products based on their nutrition were selected and screened using the agar well diffusion method. Among multiple alternatives, sugarcane juice, soya and tomato exhibited the largest zones of inhibition on the agar plates, substantiating the maximum production of rapamycin. In addition to being an excellent source of xylose and fructose, sugar cane is rich in micronutrients (Zn, P, K, Mn) that serve to enhance the production of rapamycin [24]. In the case of tomato, the abundance of tryptophan and tyrosine in addition to lysine could stimulate the shikimate pathway, which is involved in the synthesis of the rapamycin starter unit ((1R,3R,4R)-3,4 Dihydroxycyclohexanecarboxylic (DHCHC)) for rapamycin synthesis [25, 26]. Here, the present study provides the first evidence for utilizing eco-friendly, cost-effective alternatives to formulate an effective production medium for rapamycin production using *S. hygroscopicus.*

The optimum concatenation of sugarcane juice, soya and tomato was optimized using RSM to yield more production of rapamycin. The observations from the current study stated that soya and tomato are critical components and have a more significant impact than sugarcane juice. The observations indicated that the interaction between sugarcane juice and tomato was more significant than the interaction between sugarcane juice and soya. The predicted and actual values were close enough, suggesting that the model is significant. Optimizing the cost-effective medium using Box–Behnken statistical analysis of RSM increased the production by 2-fold compared with medium A. Previous studies also reported a 2-fold increase in rapamycin production in optimized media [22, 27].

The results of our study demonstrated a clear difference in the production of rapamycin in the two tested media. In medium A, rapamycin production was observed from the fourth day of incubation, with a gradual increase in concentration over time. In the initial two days, no significant cell growth or rapamycin production was recorded. Similar observations were also recorded in the RSM-optimized cost-effective medium. However, from the fourth day, a significant increase in rapamycin production was detected. For the initial three days, bothmedia showed similar growth patterns, whereas from day 4, a higher concentration of rapamycin was obtained in the cost-effective media. From day 8, the rapamycin content of the optimized medium reached 24.1 µg/mL, while that of medium A reached only11.47 µg/mL. This significant increase in rapamycin production suggests that the cost-effective medium effectively supported the growth and productivity of *S hygroscopicus*. The RSM-optimized cost-effective medium showed higher production than medium A. Additionally, the presence of amino acids in tomato, such as tryptophan, tyrosine, and lysine, in the optimized medium could have contributed to the improved production of rapamycin.

There are only two reports pointing to the use of tomato to stimulate the production of metabolites by *S. hygroscopicus*. The former was reported by Vézina et al. (1975), who incorporated tomato pulp in agar medium and obtained higher activity against *C. albicans* [1]. However, in the study, the use of tomato in formulating a production medium was not specified, nor was the production of rapamycin quantified or the medium optimized. Similarly, in a later study, enhanced growth of *S. hygroscopicus* was obtained after substituting the medium with tomato [28]. In the current study, the authors have optimized the production medium by using Box–Behnken statistical analysis of RSM. This information is valuable for the large-scale production of rapamycin, providing new insights for process optimization and maximizing yield for application in pharmaceutical industries.

## Conclusion

An optimized, cost-effective production medium for the synthesis of rapamycin by *S. hygroscopicus* was formulated. Xylose and soya peptone were identified as the best carbon and nitrogen sources respectively. Sugarcane juice and soya were used as cost-effective alternatives for xylose and soya peptone. Additionally, lysine was found to be the best amino acid for rapamycin production, which was substituted with tomato to reduce the production cost. A NaCl concentration of 1% and pH 7 were found to be most adequate for maximum production at 25 °C. The growth kinetics revealed that rapamycin production occurred between the 4th and 8th days of incubation. These findings contribute to the understanding of rapamycin production and its potential applications in industry and pharmaceutical research. The findings from the present research could potentially reduce the production cost of rapamycin, which could ultimately reduce the market price of the drug, making it more affordable and available to the public.

## Materials and methods

All media were purchased from HiMedia (India) and Merck (India). HPLC-grade solvents were purchased from Merck (Kenilworth USA, NJ). The HPLC reference standard rapamycin was from Thermo Scientific Chemicals (India).

### Microbial culture

Lyophilized *Streptomyces hygroscopicus* MTCC 1105 and the test organism *Candida albicans* MTCC were used in the current study. The *S. hygroscopicus* strain was revived in a medium containing beef extract (g/L): 12 g; peptone: 2 g; yeast extract: 2 g; tryptose: 2 g; CaCo_3_: 0.1 g; starch: 0.1 g; glucose: 10 g; CoCl_2_: 0.005 g and ferric ammonium citrate; 0.005 g. The medium was inoculated with a lyophilized seed culture of *S. hygroscopicus* and incubated at 28 °C for 7 days at pH 7.2. The test organism, *Candida albicans*, was revived using potato dextrose broth.

### Comparison of rapamycin production in different media

Three different production media were selected based on previous reports to determine the optimum medium for the production of rapamycin. Medium A (w/v) consisted of fructose: 22 g/L; mannose: 5 g/L; malt extract: 10 g/L; casein: 0.3 g/L; (NH_4_)_2_SO_4_: 5.3 g/L; NaCl: 5 g/L; K_2_HPO_4_: 4 g/L; ZnSO_4_·7H_2_O: 0.06 g/L; MgSO_4_·7H_2_O: 0.0025 g/L; MnSO_4_·H_2_O: 0.012 g/L; FeSO_4_·7H_2_O: 0.1 g/L; CoCl_2_·6H_2_O: 0.010 g/L; Na_2_SO_4_: 0.3 g/L; CaCO_3_: 3 g/L and pH 7.2. [29]. Medium B (w/v): malt extract: 3 g/L; glucose: 10 g/L; peptone: 3 g/L and pH 6.4–6.8. [30]. Medium C (w/v): dextrose, 0.5%; yeast extract, 0.25%; soluble starch, 1%; casein, 0.25%; CaCO_3_, 0.05%; pH, 7 [31]. Each medium was prepared in a 250 mL conical flask, inoculated with 2% *S. hygroscopicus* and incubated at 28 °C for 7 days. After 7 days of incubation, the cell-free supernatant was collected by centrifugation at 8000 rpm for 6 min. The quantity of rapamycin produced against *C. albicans* was determined using the well diffusion method.

### Extraction and partial purification of rapamycin

Seven-days-old *S. hygroscopicus* broth cultures were subjected to ultrasonication. Equal volumes of toluene were added to the broth culture, which was maintained at 50 °C for 4 h at 200 rpm. The amber-colored oily residue of the toluene extract was collected after 4 h, and the process was repeated. The oily residue was dissolved in equal volume of HPLC grade methanol [32].

### Determination of rapamycin production in medium A using ultra-performance liquid chromatography (UPLC)

UPLC was used to quantify the rapamycin content produced by *S. hygroscopicus* in production medium A. The concentration of rapamycin produced was determined according to Rani et al.,. Chromatographic separation was performed using a UNISON UK C18 column (3 μm diameter and 4.6 mm × 250 mm length). The run time for separation was 20 min with a flow rate of 1.0 mL/min, and a 278 nm wavelength was used to detect the compound. The mobile phase used for separation consisted of 80% solvent A (80% HPLC-grade methanol and 20% HPLC-grade acetonitrile) and 20% solvent B (HPLC-grade water). Standard rapamycin (Thermo Scientific Chemicals) was used as the reference material in the current study [32].

Rapamycin concentration was calculated using a standard curve of known rapamycin concentrations. A standard graph was calculated using different concentrations (200 µg/mL, 400 µg/mL, 600 µg/mL, 800 µg/mL, and 1 mg/mL). The peak height of the rapamycin standard was plotted against the concentration to draw a calibration graph. Linear regression was used to evaluate linearity, and repeatability was evaluated in triplicate. The amount of rapamycin produced by *S. hygroscopicus* was determined using a standard graph [33].

### Determining the time-dependent correlation of rapamycin production and anti-***Candida*** activity

Time-dependent rapamycin production by *S. hygroscopicus* was analyzed based on the zone of inhibition against *C. albicans* over time. Production medium A was inoculated with 2% of a 3-days-old culture. The inoculated cultures were incubated at 28 °C and 120 rpm. After each day, 1 mL of cell-free supernatant was collected, and the zone of inhibition was checked against *C. albicans* [21].

### Medium optimization by one factor at a time

Production medium A was optimized using different carbon (fructose, sorbitol, xylose, dextrose, sucrose, and lactose) and nitrogen sources (yeast extract, peptone, beef extract, casein, ammonium sulfate, ammonium nitrate, ammonium phosphate, ammonium chloride, potassium nitrate, and urea). Different amino acids (tyrosine, lysine, cysteine, aspartate, valine, ornithine, tryptophan, butyric acid, and serine) were used to determine the optimum amino acids for enhanced production of rapamycin. Media supplemented with different carbon sources (10 g/L), nitrogen sources (10 g/L), and amino acids (100 mg/L) were prepared separately and inoculated with 2% 3-day-old culture. All media were incubated at 20°C for 7 days at 120 rpm [30].

Temperature (15, 25, 35, 45, and 55 °C), pH (3,5,7 and 9), and NaCl concentrations (0%, 0.5%, 1%, 1.5%, and 2%) were used to optimize the medium using a one variable at a time method. Each medium was inoculated with 2% freshly prepared culture and incubated at 28 °C for 7 days at 120 rpm. After 7 days of incubation, cell-free supernatants were collected from each medium, and the zone of inhibition against *C. albicans was determined*. All experiments were performed in triplicate [30].

### Cost effective medium

Based on one factor at a time, the cheapest sources of xylose, soya peptone, and lysine were selected. In medium A, xylose was replaced by straw from rice, bagasse, sugar cane juice, and corncobs, and the nitrogen source soya peptone was replaced with dried leaves of *Pisum sativum*, grass, and soya bean powder. Dried tomatoes were used as a source of lysine. Each medium was prepared separately and inoculated with 2% freshly prepared cultures. All media were incubated at 25 °C for 7 days at 120 rpm. The cell-free supernatant was collected on the 7^th^ day, and the zone of inhibition against *C. albicans* was determined.

### Optimization of cost-effective medium using response surface methodology

The effects of sugarcane, soya, and tomatoes were estimated using the Box–Behnken statistical method. This tool was used to estimate the effects of individual and mutual interactions among sugarcane, soya, and tomatoes on the production of rapamycin. The Box–Behnken design was generated using Design-Expert 13 software (StatEase Inc., Minneapolis, MN, United States). The low, medium, and high values of each component were as follows: sugarcane (mL/L), 100, 150, and 200; tomato (g/L), 5, 12.5, and 20; soya (g/L), 5, 12.5, and 20. A total of 17 runs were generated, and all media were prepared in 50 mL. A 2% fresh culture was inoculated in the media and incubated at 25 °C for 7 days at 120 rpm. The production medium was subjected to toluene extraction to attain partially purified rapamycin. The amount of rapamycin produced in each medium was estimated by UPLC. All runs were performed in triplicate, and the mean value was used for the RSM analysis. Rapamycin production (response 1) was the dependent variable and was statically validated using ANOVA [34].

### Growth kinetics of ***S. hygroscopicus*** in medium A and cost-effective medium

The production rate of rapamycin by *S. hygroscopicus* was analyzed based on UPLC analysis. Production medium A and optimized cost-effective medium were inoculated with 2% of a 3-days-old culture. The inoculated cultures were incubated at 28 °C and 120 rpm. Cell growth and rapamycin production were checked from the 1^st^ to 8^th^ day of incubation. All experiments were performed in triplicates [21].

### Electronic supplementary material

Below is the link to the electronic supplementary material.

Supplementary Material 1

## Data Availability

All data generated or analysed during this study are included in this article.
