# Supplementary material for: Formulation of cost-effective medium and optimization studies for enhanced production of rapamycin
Source: Microb Cell Fact. 2023 Sep 20;22:189. doi: 10.1186/s12934-023-02201-3 (PMC10510133; doi:10.1186/s12934-023-02201-3)
Supplement: Supplementary file 1 — Supplementary Material 1 [file 12934_2023_2201_MOESM1_ESM.docx]

**SUPPLEMENTARY DATA**

**Formulation of cost-effective medium and optimization studies for enhanced production of rapamycin**

Sanjeev. K. Ganesh, C. Subathra Devi*

School of Bio Sciences and Technology, Vellore Institute of Technology, Vellore-632014, Tamil Nadu, India.

***Correspondence**: C. Subathra Devi , Associate Professor senior, Department of Biotechnology, School of Bio Sciences and Technology, Vellore Institute of Technology, Vellore- 632 014, Tamil Nadu, India

**Email**: csubathradevi@vit.ac.in, subaresearch@rediffmail.com

**Phone**: +91-9486420509

**Fax**: +91-4162243092


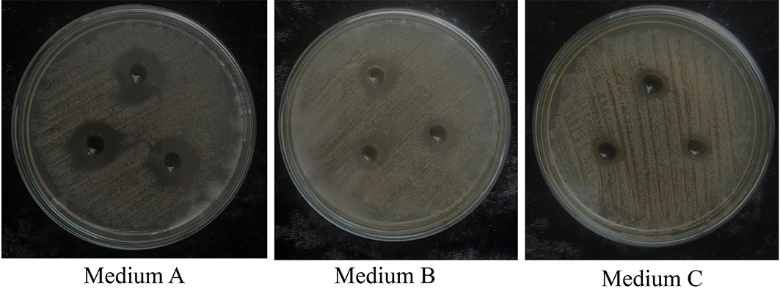


Figure: S1: Anti candida activity of rapamycin extracted from medium A, medium B and medium C
